# Does cognitive impairment impact adherence? A systematic review and meta-analysis of the association between cognitive impairment and medication non-adherence in stroke

**DOI:** 10.1371/journal.pone.0189339

**Published:** 2017-12-08

**Authors:** Daniela Rohde, Niamh A. Merriman, Frank Doyle, Kathleen Bennett, David Williams, Anne Hickey

**Affiliations:** 1 Division of Population Health Sciences, Royal College of Surgeons in Ireland, Dublin, Ireland; 2 Department of Geriatric and Stroke Medicine, Beaumont Hospital and Royal College of Surgeons in Ireland, Beaumont Hospital, Dublin, Ireland; University of Glasgow, UNITED KINGDOM

## Abstract

**Background:**

While medication adherence is essential for the secondary prevention of stroke, it is often sub-optimal, and can be compromised by cognitive impairment. This study aimed to systematically review and meta-analyse the association between cognitive impairment and medication non-adherence in stroke.

**Methods:**

A systematic literature search of longitudinal and cross-sectional studies of adults with any stroke type, which reported on the association between any measure of non-adherence and cognitive impairment, was carried out according to PRISMA guidelines. Odds ratios and 95% confidence intervals were the primary measure of effect. Risk of bias was assessed using the Cochrane Bias Methods Group's Tool to Assess Risk of Bias in Cohort Studies, with evidence quality assessed according to the GRADE approach. We conducted sensitivity analyses according to measure of cognitive impairment, measure of medication adherence, population, risk of bias and adjustment for covariates. The protocol was registered with PROSPERO.

**Results:**

From 1,760 titles and abstracts, we identified 9 studies for inclusion. Measures of cognitive impairment varied from dementia diagnosis to standardised cognitive assessments. Medication adherence was assessed through self-report or administrative databases. The majority of studies were of medium risk of bias (n = 6); two studies had low risk of bias. Findings were mixed; when all studies were pooled, there was no evidence of an association between cognitive impairment and medication non-adherence post-stroke [OR (95% CI): 0.85 (0.66, 1.03)]. However, heterogeneity was substantial [*I*^2^ = 90.9%, *p* < .001], and the overall evidence quality was low.

**Conclusions:**

Few studies have explored associations between cognitive impairment and medication adherence post-stroke, with substantial heterogeneity in study populations, and definitions and assessments of non-adherence and cognitive impairment. Further research using clear, standardised and objective assessments is needed to clarify the association between cognitive impairment and medication non-adherence in stroke.

## Introduction

Secondary prevention is essential to maximising health and wellbeing post-stroke. Recurrent strokes account for up to a third of all strokes [1], and are associated with significantly increased risks of mortality [2], long-term disability [3], and dementia [4]. Controlling vascular risk factors through secondary prevention medications, including lipid-lowering medications, antihypertensive and antithrombotic treatment, is vital to decreasing the risk of stroke recurrence [5–8]. Effective secondary stroke prevention is contingent on consistent adherence to prescribed secondary preventive medications [9]. However, medication adherence is frequently poor, with a non-adherence estimate of 30.9% (95% CI: 26.8, 35.3) reported for patients following stroke or transient ischaemic attack (TIA) [10]. Non-adherence is associated with adverse outcomes, including rehospitalisation, recurring vascular events, and death, as well as increased costs of care [8, 11, 12]. Medication adherence has been proposed to consist of three phases: patient initiation, implementation, and discontinuation (non-persistence) [13]. Non-adherence can thus be defined as a patient’s failure to initiate prescribed therapy, sub-optimal implementation of a medication regimen, or early, non-physician initiated discontinuation or non-persistence [13]. We applied this broad definition in order to capture the full breadth of the non-adherence literature.

### Cognitive impairment and medication non-adherence

Stroke is associated with a close to 2-fold increased risk of cognitive decline [14], while existing cognitive impairment may predispose to stroke [15, 16]. Cognitive impairment can further increase disability and levels of dependency in patients with stroke, leading to greater burden on carers and the healthcare system [15]. Medication taking involves several cognitive functions, including accessing and scheduling medications, and understanding, remembering and following instructions, all of which may be affected by cognitive impairment [17]. Cardiovascular risk factors, such as hyperlipidaemia, hypertension, and diabetes increase the risk of cognitive decline and dementia [18–20]. The use of anticoagulant, antiplatelet and antihypertensive medications has been reported to be associated with a reduced risk of cognitive impairment post-stroke [7], suggesting that optimum control of risk factors through the regular use of cardiovascular medications could reduce the risk of cognitive decline, as well as reducing the risks of recurrent stroke and cardiovascular events [17, 21].

Considering the prevalence of poor adherence, it is important to consider whether or not patients actually take their medications when evaluating the impact of medications on outcomes [22]. Cognitive impairment has been reported to be associated with poorer adherence to medications in asymptomatic carotid stenosis [21], heart failure [23, 24], and general older adult samples [25, 26], while a recent systematic review explored medication non-adherence in community-dwelling persons with dementia and cognitive impairment [27]. However, only a small number of studies have explored associations between cognitive impairment and adherence post-stroke, with discordant results. Two recent systematic reviews examining a variety of factors associated with medication adherence in stroke featured only a small number of studies of cognitive or memory impairments, and did not meta-analyse the results [10, 28]. The aim of this study, therefore, was to systematically review and meta-analyse the association between cognitive impairment and medication non-adherence in patients with stroke.

## Materials and methods

### Study design

We performed a systematic review and meta-analysis according to PRISMA guidelines [29]. The review protocol was registered with PROSPERO (available from http://www.crd.york.ac.uk/PROSPERO/display_record.asp?ID=CRD42015027316).

### Eligibility criteria

#### Study designs

Both longitudinal (cohort and (non)randomised controlled trials) and cross-sectional studies were eligible for inclusion. Published abstracts were included if missing information was available from the authors. We excluded qualitative studies, reviews, letters, editorials, and discussion papers.

#### Participants

Studies of adults aged ≥18 years with any stroke type (ischaemic or haemorrhagic, first or recurrent) were eligible. Patients with TIA were included. Studies were excluded if the study population was <18 years. Studies that included general patient populations were eligible for inclusion if sub-group analyses were available for patients with stroke. Studies that assessed cognitive impairment and adherence as either exposure or outcome at any time point (baseline or follow-up) were included.

#### Cognitive impairment

Cognitive impairment can range from mild dysfunction to dementia; therefore, studies reporting any measure of cognitive impairment, including a diagnosis of dementia or standardised cognitive assessment, were included [17].

#### Adherence

Studies reporting any measure of medication (non)adherence or (non)persistence by patients, such as self-report, pill counts, or pharmacy prescription refill data, were included [13, 17]. Studies that did not specify how adherence was assessed were excluded.

### Search methods and information sources

The following electronic databases were searched without language restrictions from database start to 31^st^ December 2016: PubMed, EMBASE, PsycINFO, Web of Science, Scopus, Cochrane Library. Search strategies were developed in consultation with a subject librarian. Search terms included variations and synonyms of stroke, cognitive impairment, adherence, and medication. Search strategies for all databases are presented in the Supporting Information (S1 Table). We augmented searches with reference and Google Scholar citation searches of included studies.

### Data collection and analysis

#### Screening and extraction

Retrieved records were imported to Covidence. Two reviewers (DR and NAM/AH) independently screened titles and abstracts to identify studies potentially meeting the inclusion criteria. Disagreements were resolved through discussion. Full texts of potentially eligible studies were retrieved and assessed for eligibility by the first author. Study authors were contacted for missing data or further information as necessary.

Data were extracted by the first author using a standardised form, including: authors, study design, sample size, sample description, length of follow-up, measure of medication adherence, measure of cognitive impairment, results, and conclusions.

#### Risk of bias

As all included studies were either cohort studies (n = 8) or based on secondary analysis of RCTs (n = 1), we assessed risk of bias using the Cochrane Bias Methods Group's Tool to Assess Risk of Bias in Cohort Studies [30]. This checklist assesses risk of bias, from low to high, for sample selection, assessment of exposure and outcome, presence/absence of outcome at the beginning of the study, adjustment for prognostic variables, and follow-up. Two reviewers (DR and NAM) independently assessed risk of bias, with disagreements resolved through discussion. Due to the small number of studies identified, we did not exclude any studies based on risk of bias, but instead conducted a sensitivity analysis based on risk of bias.

#### Evidence quality

The overall quality of the evidence was assessed using the Grading of Recommendations, Assessment, Development, and Evaluation (GRADE) approach, which evaluates study design, study quality, consistency, and directness [31]. Using this approach, observational studies are initially assigned a low grade of evidence, but can be upgraded if there is evidence of a strong and consistent association with no plausible confounders or threats to validity, evidence of a dose response gradient, or when all plausible confounders would have reduced the observed effect [31]. Studies are downgraded for risk of bias, inconsistency of results, indirectness of evidence, imprecision, or publication bias [32]. Potential publication bias was explored by means of a funnel plot.

### Data analysis

We conducted a narrative synthesis and meta-analysis. The majority of included studies reported odds ratios (ORs) or hazard ratios (HRs) as measure of effect of the association between cognitive impairment and medication non-adherence. In order to facilitate quantitative pooling of all studies, extracted results for the remaining studies were converted to ORs and 95% confidence intervals (CIs), using 2x2 tables or effect size conversion calculators [33–35]. We conducted a random-effects meta-analysis using the metan command in Stata^®^ 13.0, with heterogeneity assessed using *I*^2^. For studies that reported associations between cognitive impairment and adherence at numerous time points, we included results pertaining to the longest follow-up period. Where available, we used adjusted results. Given the significant heterogeneity between studies, sensitivity analyses were conducted according to measure of cognitive impairment (dementia diagnosis vs. standardised cognitive assessment), measure of adherence (objective assessments vs. self-report), adjustment for covariates (adjusted vs. unadjusted), risk of bias, and population (participants with atrial fibrillation (AF) vs. all others, due to preponderance of focus on anticoagulant adherence in included studies).

## Results

### Study selection

**Fig 1:**
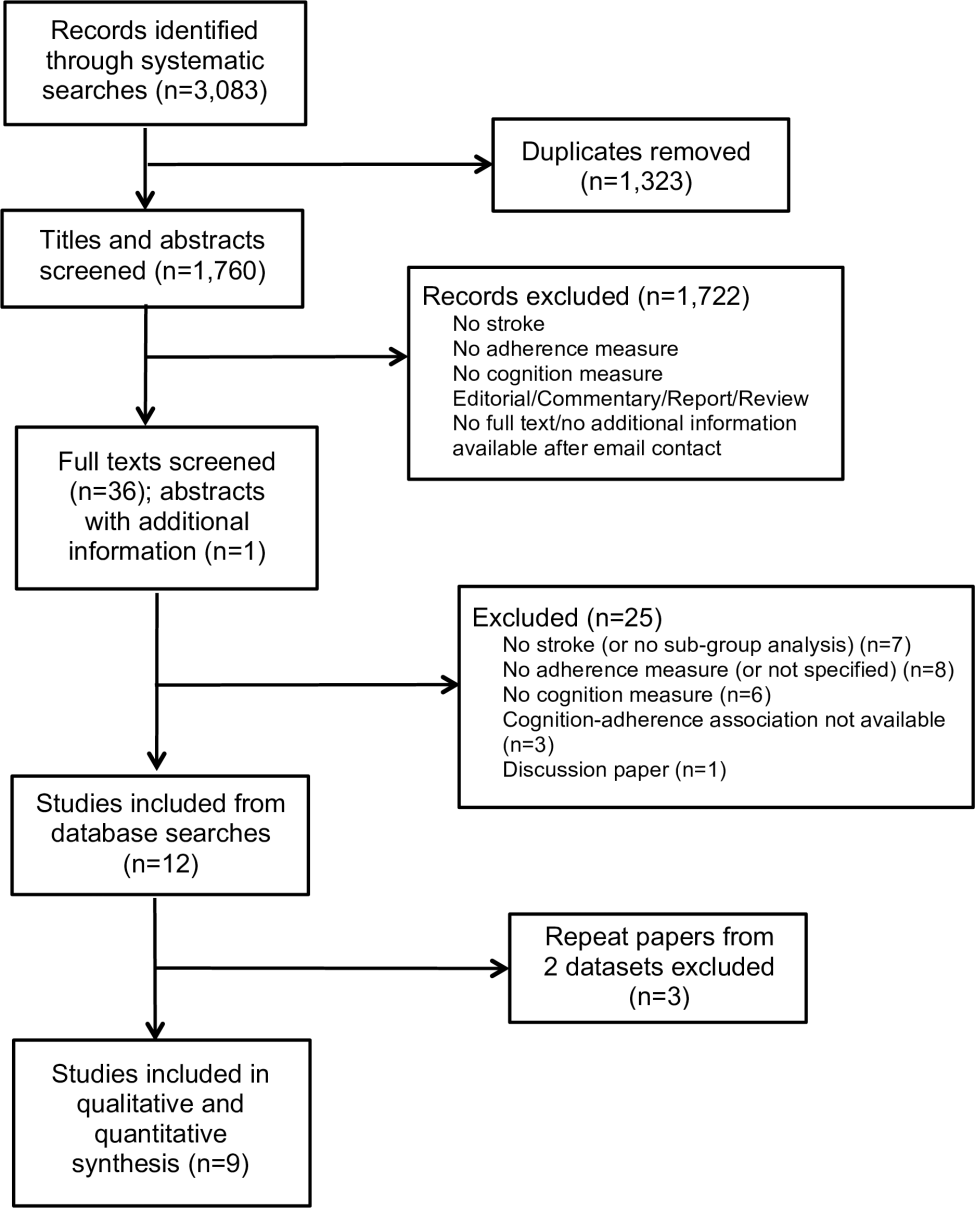
Flow chart of included studies.

The searches returned 3,083 records, including 1,323 duplicates, resulting in 1,760 titles and abstracts screened for inclusion. 1,722 records were excluded following title and abstract screening; reasons for exclusion are detailed in Fig 1. This left 36 papers for full text screening, and one published abstract with missing information provided by the authors. Following full text screen, 24 papers were excluded. Three repeat papers from two datasets were also excluded, resulting in 9 included studies [36–44].

### Study characteristics

#### Design

The majority of included studies were retrospective (n = 4) [36, 41–43] or prospective cohort studies (n = 4) [37–40]. One study was based on secondary analysis from the Secondary Prevention of Small Subcortical Strokes trial [44]. Length of follow-up ranged from 5–6 weeks to 3 years, with results of one study based on cross-sectional analysis (Table 1). All studies reported a measure of medication (non)adherence or persistence as the outcome, with a measure of cognitive impairment as exposure.

**Table 1:** Characteristics of included studies.

Author, Year, Location	Design	N (baseline, follow-up)	Follow-up	Population	Outcome	Adherence measure	Cognitive impairment measure	Statistical results	Effect sizes for meta-analysis (Cognitive impairment and non- adherence)	Adjusted for	RoB
Björck 2015 Sweden [36]	Retrospective cohort	4583, 4583	up to 5 years	Stroke and AF	Warfarin non- persistence (treatment gap >7 days registered in AuriculA)	AuriculA: Swedish national quality register for AF and OAC (OAC use registered and updated daily)	Dementia diagnosis (Swedish National Patient Register) (n = 45)	Patients with dementia more likely to be non-persistent [HR 2.22 (1.51, 3.27)].	OR (95% CI): 3.0593 (0.6796, 5.5722)		Low
Gumbinger 2015 Germany [39]	Prospective cohort	284, 139	15 months	IS/TIA and AF	Anticoagulant adherence	Self-report (ascertained through interview)	Dementia diagnosis (stated by the patient, the primary care physician, or relatives) (n = 17)	Dementia predicted non-adherence [OR 18.01 (2.11, 153.25)].	OR (95% CI): 18.01 (2.11, 153.25)	Sex, nursing home residence	Medium
Shah 2016 Canada [41]	Retrospective cohort	2877, 2877	12 months	IS/TIA and AF	Oral anticoagulant adherence	Prescription claims (PDC<0.4 = poor adherence)	Dementia diagnosis (Ontario Stroke Registry, based on hospital chart review) (n = 590)	Dementia not associated with poor adherence (PDC <0.4) [OR 1.26 (0.77, 2.04)].	OR (95% CI): 1.26 (0.77, 2.04)	Age, sex, income, TIA/stroke, residence, stroke severity, comorbidities, long-term care residence.	Low
Wawruch 2016a Slovakia [43]	Retrospective cohort	4319, 4319	3 years	Stroke	Antiplatelet non-persistence	Prescription records (treatment gap> = 6 months)	Dementia diagnosis (extracted from the database of the largest health insurance provider in the Slovak Republic) (n = 694)	Dementia decreased probability of non-persistence [HR 0.69 (0.57, 0.83)].	OR (95% CI): 0.526 (0.428, 0.648)	Age, sex, hypertension, diabetes, high cholesterol, depression, anxiety, Parkinson's, epilepsy, polypharmacy, medication switching.	Medium
Wawruch 2016b Slovakia [42]	Retrospective cohort	2748, 2748	3 years	Ischaemic stroke	Statin non-persistence	Prescription records (treatment gap> = 6 months)	Dementia diagnosis (extracted from the database of the largest health insurance provider in the Slovak Republic) (n = 518)	Dementia decreased probability of non-persistence [HR 0.84 (0.73, 0.98)].	OR (95% CI): 0.6366 (0.5129, 0.7901)	Age, sex, hypertension, diabetes mellitus, hypercholesterolemia, depression, anxiety.	Medium
Coetzee 2008 Australia [38]	Prospective cohort	Baseline unclear, 25	6 weeks	IS/TIA	Medication adherence	Pill counts and self-report (Treatment Assessment Schedule)	Cognitive assessment (EFQ)	Memory dysfunction associated with poorer adherence [*r* = -.54]. Dysfunction in planning/ organisation associated with poorer adherence [*r* = -.52].	OR (95% CI): 10.248 (2.154, 49.029)		High
O'Carroll 2011 Scotland [40]	Prospective cohort	180, 180	5–6 weeks	Ischaemic stroke	Medication adherence	Self-report (MARS)	Cognitive assessment (MMSE)	Cross-sectional—MMSE score associated with adherence score (β = 0.201).		Age, sex, stroke severity, illness perception and belief about medications variables, emotional distress, social deprivation index, perception of risk of further stroke.	Medium
								Longitudinal- MMSE score not associated with adherence score (β = 0.005).	OR (95% CI): 1.000 (0.589, 1.698)		
White 2010 North America, Latin America, Spain [44]	RCT (patients active on both arms included)	526, 471, 323	3 years	Lacunar stroke	Medication adherence	Pill counts and self-report (self-report method unclear)	Cognitive assessment (CASI)	No association between cognitive impairment and adherence at year 1 [OR 1.001 (0.981, 1.021)]; year 2 [OR 0.988 (0.960, 1.016)]; year 3 [OR 0.979 (0.933, 1.028)].	OR (95% CI): 1.021 (0.973, 1.071)	Age, sex, education, ethnicity, employment status, smoking, alcohol consumption, exercise, BMI, marital status, living arrangements, health rating, number of medications, Rankin, Barthel, missed clinic visits, previously inactive on any therapy.	Medium
Brewer 2015 Ireland [37]	Prospective cohort	302, 256	6 months	Ischaemic stroke	Medication adherence	Self-report (MARS)	Cognitive assessment (MoCA)	Cross-sectional analysis: absence of cognitive impairment associated with non-adherence [OR 1.10 (1.04, 1.17)].	OR (95% CI): 0.91 (0.85, 0.96)	Age, sex.	Medium

**Note:** IS ischaemic stroke; PDC proportion of days covered; RoB risk of bias; OAC oral anticoagulant

#### Population

Three studies included stroke or stroke and TIA patients with AF [36, 39, 41]. Five studies included ischaemic stroke or ischaemic stroke and TIA patients [37, 40, 42–44], one study included mixed stroke types [38]. Sample sizes ranged from 25 to 4,583 participants.

#### Cognitive impairment measure

Five studies included a diagnosis of dementia as a measure of cognitive impairment [36, 39, 41–43], while one study each used the Montreal Cognitive Assessment (MoCA) [37], Mini Mental State Examination (MMSE) [40], Cognitive Abilities Screening Instrument (CASI) [44], or Everyday Functioning Questionnaire (EFQ) [38]. Diagnosis of dementia was based on data recorded in health insurance or patient registry databases [36, 41–43], or on report by the patient, relative, or primary care physician [39].

#### Outcome measure

Assessments of (non)adherence included the Medication Adherence Report Scale (MARS) (n = 2) [37, 40], self-report either alone or in combination with pill counts (n = 3) [38, 39, 44], prescription records (n = 3) [41–43], or ongoing treatment registered in a Swedish national quality register for atrial fibrillation and oral anticoagulation (AuriculA) (n = 1) [36]. Three studies considered adherence to anticoagulant medications in stroke patients with AF [36, 39, 41], one study each focused on antiplatelets [43] and statins [42]. Three studies utilising self-report did not distinguish between medications [37, 38, 40], while one study combined pill counts of antiplatelet medications with self-reported antihypertensive medication adherence to create a composite adherence measure [44]. In addition to the MARS, self-report measures of adherence included the non-validated Treatment Assessment Schedule [38]. Two studies did not provide details on the use of self-report instruments [39, 44].

#### Risk of bias

The majority of studies were rated at medium risk of bias [37, 39, 40, 42–44], with two studies considered at low [36, 41] and one at high risk of bias [38].

#### Evidence quality

**Fig 2:**
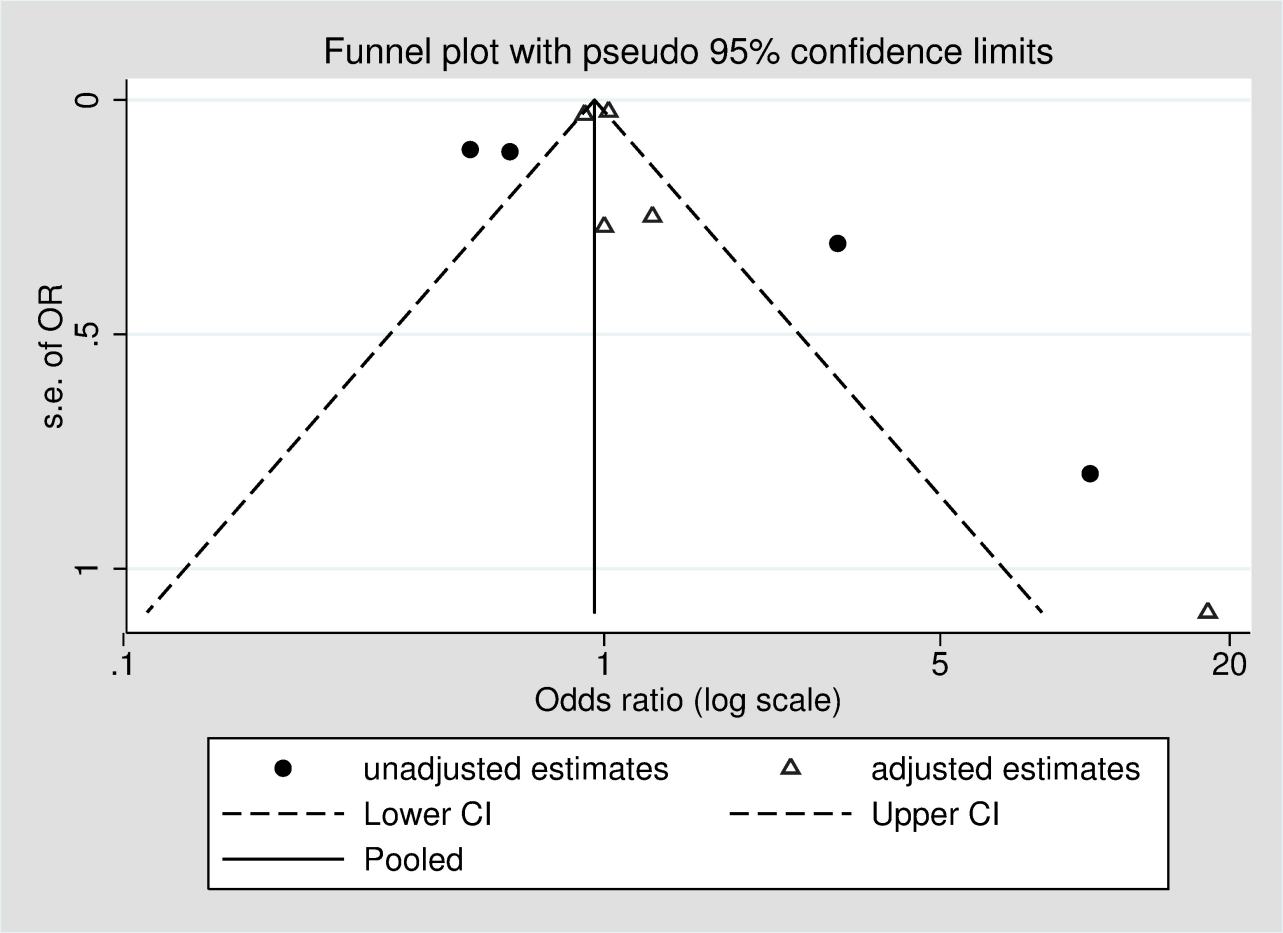
Funnel plot of included studies, stratified by adjustment for covariates.

Based on the GRADE approach, the overall quality of the evidence was rated as low, with no studies being upgraded from their initial rating, and two studies downgraded due to inconsistent large and imprecise effect estimates based on unadjusted or minimally adjusted analyses and uncertainty about the directness of the predictor or outcome (Table 2). Fig 2 displays a funnel plot of included studies, showing a potential absence of small and medium-sized studies, and smaller studies reporting that cognitive impairment may reduce the likelihood of medication non-adherence. However, it is important to note that asymmetry in funnel plots can have several causes, including heterogeneity or chance [45]. The pseudo 95% confidence limits indicate the distribution of studies that would be expected in the absence of heterogeneity [46], indicating that the asymmetry seen here could be due to significant heterogeneity between studies, for example as a result of differences between adjusted and unadjusted estimates.

**Table 2:** GRADE quality of evidence.

	Quality assessment					Summary of findings		
Studies	Design	Risk of bias	Consistency	Directness	Other modifying factors	No of participants at follow-up	Effect OR (95% CI)	Quality of evidence (GRADE)
**Cognitive assessment as measure of cognitive impairment**								
Brewer [37]	Observational	Medium	No major inconsistencies	Direct (but self-report)	Cross-sectional analysis	256	0.91 (0.85, 0.96)	Low ⊕⊕
O’Carroll [40]	Observational	Medium	No major inconsistencies	Direct (but self-report)		180	1.000 (0.589, 1.698)	Low ⊕⊕
White [44]	RCT (analysis of adherence based both active arms)	Medium	No major inconsistencies	Uncertainty about outcome measure–unclear self report method		323	1.021 (0.973, 1.071)	Low ⊕⊕
Coetzee [38]		High	Effect estimate is large and imprecise.	Uncertainty about directness of predictor–cognitive impairment based on memory dysfunction or dysfunction in planning/organisation (rather than global cognitive impairment). Uncertainty about outcome measure–non-validated self-report	Estimate is based on unadjusted analyses.	25	10.248 (2.154, 49.029)	Very low ⊕
**Dementia diagnosis as measure of cognitive impairment**^**$**^								
Gumbinger [39]	Observational	Medium	Effect estimate is large and imprecise	Uncertainty about outcome measure–non-validated self-report	Minimal adjustment for confounders.	139	18.01 (2.11, 153.25)	Very low ⊕
Björck [36]	Observational	Low	Effect estimate is quite large.		Estimate included in meta-analysis is unadjusted.	4583	3.059 (0.680, 5.572)	Low ⊕⊕
Shah [41]	Observational	Low	No major inconsistencies	PDC<0.4 taken to indicate poor adherence–more usual to use cut-off of <0.8		2877	1.26 (0.77, 2.04)	Low ⊕⊕
Wawruch a [43]	Observational	Medium	No major inconsistencies		Estimate included in meta-analysis is unadjusted	4319	0.526 (0.428, 0.648)	Low ⊕⊕
Wawruch b [42]	Observational	Medium	No major inconsistencies		Estimate included in meta-analysis is unadjusted	2748	0.637 (0.513, 0.790)	Low ⊕⊕

Note: observational studies are assigned a baseline rating of low in the GRADE system. Studies may be upgraded if there is a large magnitude of effect, evidence of a dose response relationship, or when all plausible confounders would have reduced the observed effect

^$^ Some uncertainty about directness of predictor. Diagnosis of dementia represents the severe end of the cognitive impairment spectrum only. Several studies have reported physician-initiated discontinuation of anticoagulants in patients with dementia, which may confound associations between dementia and adherence to anticoagulants (considered by Gumbinger, Björck and Shah).

### Associations between cognitive impairment and medication non-adherence

**Fig 3:**
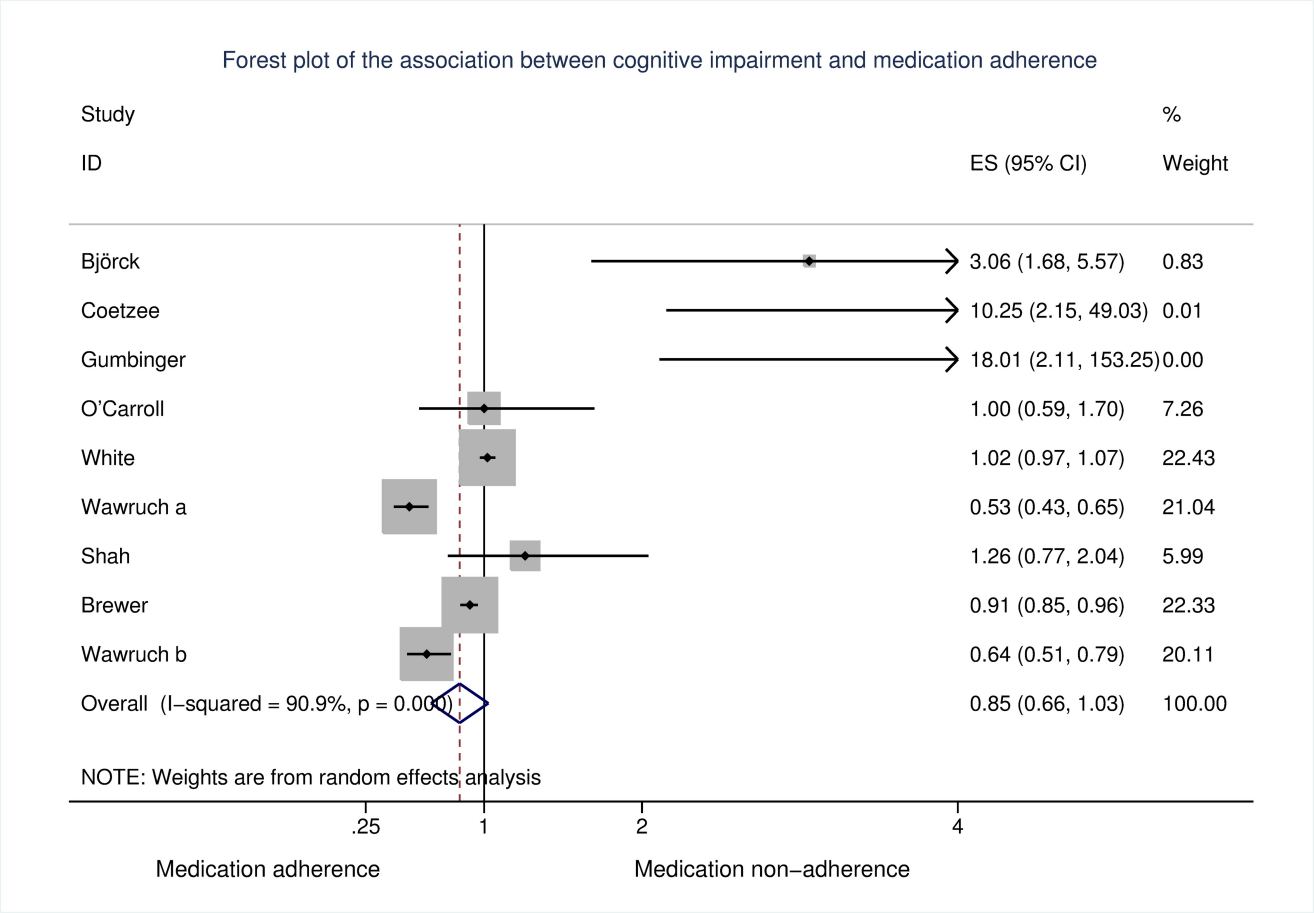
Forest plot of included studies.

Evidence on the association between cognitive impairment and non-adherence was discordant; three studies found no association between medication adherence and cognitive impairment [40, 41, 44], three reported that cognitive impairment was associated with increased non-adherence [36, 38, 39] while three studies reported that cognitive impairment was associated with decreased non-adherence [37, 42, 43]. When all studies were pooled, there was no evidence of an association between cognitive impairment and non-adherence [OR (95% CI): 0.85 (0.66, 1.03)]; however, heterogeneity was substantial [*I*^2^ = 90.9%, *p* < .001] (Fig 3). Excluding one study rated at high risk of bias did not affect this estimate [OR (95% CI): 0.85 (0.66, 1.03); *I*^*2*^ = 92.0%, *p* < .001]. Due to the significant heterogeneity between studies and differing study populations, assessments of cognitive impairment and adherence, and adjustment for covariates, a number of sensitivity analyses were conducted.

### Sensitivity analyses

#### Measure of cognitive impairment

Five studies included a diagnosis of dementia as the measure of cognitive impairment [36, 39, 41–43]. Two of these reported increased non-adherence in patients with dementia [36, 39], while two reported that dementia was associated with reduced non-adherence [42, 43]. When these five studies were pooled, diagnosis of dementia appeared to be associated with a reduced likelihood of non-adherence [OR (95% CI): 0.70 (0.45, 0.94)]; however, there was significant heterogeneity between studies [*I*^2^ = 67.7%, *p* = .015]. Each of the remaining four studies used a different assessment of cognitive impairment. When these studies were pooled, there was no evidence of an association between cognitive impairment and medication adherence [OR (95% CI): 0.97 (0.87, 1.07); *I*^2^ = 67.9%, *p* = .025] (Table 3).

**Table 3:** Meta-analysis and sensitivity analyses.

Included studies		Medication non-adherence	
		OR (95% CI)	Heterogeneity (*I*^2^)
All		0.845 (0.664, 1.026)	90.9%
Measure of cognitive impairment	Diagnosis of dementia (n = 5)	0.696 (0.451, 0.942)	67.7%
	Assessment of cognitive impairment (n = 4)	0.968 (0.870, 1.065)	67.9%
Population	Stroke patients with AF only (n = 3)	0.827 (0.436, 3.219)	36.5%
	All other stroke patients (n = 6)	0.799 (0.614, 0.983)	93.9%
Adherence measure	Objective (n = 4)	0.703 (0.448, 0.958)	75.4%
	Self-report (n = 5)	0.968 (0.875, 1.060)	58.0%
Adjustment for covariates	Adjusted (n = 4)	0.973 (0.881, 1.066)	58.8%
	Unadjusted (n = 5)	0.612 (0.390, 0.834)	64.4%
Risk of Bias	Medium (n = 6)	0.798 (0.614, .983)	93.9%
	Low (n = 2)	1.915 (0.218, 3.612)	66.3%
	High (n = 1)	10.248 (2.154, 49.029)	

#### Study population

Three studies included stroke or stroke/TIA patients with AF, and assessed adherence to anticoagulant medications [36, 39, 41]. All three studies included a diagnosis of dementia, but used a different measure and definition of adherence. Two studies noted that non-adherence was more likely in patients with dementia [36, 39]; however, when all three were pooled, there was no evidence of an association between cognitive impairment and non-adherence [OR (95% CI): 1.83 (0.44, 3.22); *I*^2^ = 36.5%, *p* = 0.207]. The remaining studies did not focus exclusively on patients with stroke and AF. When these studies were subjected to meta-analysis, cognitive impairment appeared to be associated with a reduced likelihood of non-adherence; however, heterogeneity between studies was substantial [OR (95% CI): 0.80 (0.61, 0.98); *I*^2^ = 93.9%, *p* < .001].

#### Measure of medication adherence

When studies that assessed medication adherence based on administrative databases (prescription claims or national register) were pooled, dementia again appeared to be associated with a reduced likelihood of non-adherence [OR (95% CI): 0.70 (0.45, 0.96); *I*^2^ = 75.4%, *p* = .007]. Conversely, there was no evidence of an association between medication non-adherence based on self-report (either alone or in combination with pill counts), and cognitive impairment OR (95% CI): 0.97 (0.88, 1.06); *I*^2^ = 58.0%, *p* = .049].

#### Adjustment for covariates

Adjustment for covariates varied widely between studies (Table 1). While we included adjusted measures of effect size in the meta-analysis where possible, in order to facilitate pooling of estimates from all studies, some unadjusted results were included [36, 38, 42, 43]. When studies with adjusted and unadjusted estimates were considered separately, there was no evidence of an association between cognitive impairment and medication non-adherence in studies with adjusted results [OR (95% CI): 0.97 (0.88, 1.07); *I*^2^ = 58.8%, *p* = .046]. However, for studies with unadjusted estimates, cognitive impairment was associated with reduced non-adherence [OR (95% CI): 0.61 (0.39, 0.83); *I*^2^ = 64.4%, *p* = .038]. The funnel plot suggests that these differences between studies reporting adjusted and unadjusted results may partially account for the heterogeneity between studies (Fig 2).

#### Adjustment for long-term care residence, living arrangements or social support

The majority of studies did not include information on living arrangements, long-term care residence or social support, all of which may plausibly influence adherence. Two studies controlled for long-term care residence in their analyses of the association between cognitive impairment and non-adherence. Gumbinger et al. reported that nursing home residence was a risk factor for non-adherence to oral anticoagulants [39], while Shah et al. found no association between long-term care residence and non-adherence in adjusted analyses [41]. White et al. adjusted for living arrangements (alone vs. with others), and found no evidence of an association between living arrangements and adherence [44]. Coetzee et al. reported that social support was associated with better adherence in unadjusted analyses [38].

## Discussion

When all studies were pooled, we found no evidence of an association between cognitive impairment and medication non-adherence. The substantial heterogeneity in study populations and various definitions and assessments of adherence and cognitive impairment, combined with the overall low quality of the evidence, make it difficult to draw definitive conclusions. Significant heterogeneity was also noted in two recent systematic reviews on adherence to secondary preventive medications post-stroke [10, 28]. It may be that no association exists between cognitive impairment and medication adherence, with associations reported by observational studies due to inadequate adjustment for confounding. Indeed, while cognitive impairment was associated with reduced medication non-adherence in studies reporting unadjusted results, we found no association between cognitive impairment and adherence in our sensitivity analysis of studies reporting adjusted results. A recent study of a general adult population similarly found no association between cognitive impairment and adherence to cardiovascular medications after adjustment for a range of potential confounders [22].

It may, however, be important to distinguish between degrees of cognitive impairment, as individuals with more severe impairments and dementia may rely on caregivers to administer medications, leading to increased adherence [17, 47], while those with mild cognitive impairment managing their own medications may be most at risk of sub-optimal adherence. The association between cognitive impairment and adherence, if it exists, may in fact be U-shaped, with poorer adherence in patients with milder cognitive impairments who self-administer their medications, and better adherence in patients with more severe impairments who receive support with medication taking. Increased support from family and higher levels of care at home have been reported to be associated with better adherence [27, 28]. The majority of included studies did not report living arrangements, long-term care residence or social support, with no clear pattern emerging regarding the potential impact of these factors on adherence in studies that did include them. Indeed, there is limited information on factors associated with non-adherence in individuals who rely on family members or carers for medication management, and further research in this area is required [48, 49]. While we found that a diagnosis of dementia may be associated with better medication adherence, there was no evidence of an association for cognitive impairment based on cognitive assessments. However, we were unable to distinguish between degrees of cognitive impairment in these studies. A diagnosis of dementia has also been associated with physician-initiated discontinuation of oral anticoagulation in stroke patients (for example, due to a perceived increased risk of falls) [39, 41, 50], further complicating the association between medication adherence and cognitive function post-stroke.

A substantial number of studies screened for this review did not include or report measures of cognitive impairment. A smaller number assessed both cognitive impairment and medication non-adherence, but did not report the association between these two measures; or included general adult/patient populations and did not report sub-group analyses for stroke survivors (Supporting Information S2 Table). The authors of these studies were contacted for further information; however, data were either unavailable (n = 4) or no response was received (n = 4), a problem noted in other systematic reviews [28]. Few studies to date have explored or reported associations between cognitive impairment and adherence post-stroke, with six of the nine studies included in this review published since 2015.

A variety of factors can influence stroke patients’ medication adherence, including concerns about treatment, knowledge about medications and beliefs about benefits and consequences [28, 40], increased disability, more severe stroke, polypharmacy [10], living in a nursing home and initiation of medications during in-hospital stay [39], self-rated health [44], age [40, 41], sex [43, 51], education [51], and presence of other comorbidities [36]. Considering the number of different measures and definitions of adherence and cognitive impairment, it is not surprising that predictors of medication (non)adherence have varied widely between studies. The use of a similarly wide variety of definitions and measures of medication non(adherence) has been noted by other systematic reviews [27, 52]. This lack of conceptual clarity in the definition and measurement of adherence leads to difficulties in comparing methods and results across studies [52]. While sensitivity analyses suggested a potential association between cognitive impairment and adherence based on objective measures, there was no association between self-reported adherence and cognitive impairment. Self-report instruments are subject to social desirability and recall bias, which may be particularly problematic in patients with cognitive impairments [12]. Where possible, future studies should focus on objective measures, such as prescription records, to assess adherence. Future studies could also explore how adherence to secondary preventive medications might affect post-stroke cognitive impairment or decline [49], and investigate associations between medication adherence and individual cognitive domains [27].

### Limitations

Only published, peer-reviewed articles or published abstracts of conference proceedings were considered for this review. Due to time and resource constraints, grey literature was excluded, which may lead to a publication or time lag bias. While two reviewers independently screened titles and abstracts, full text screening and data extraction were conducted by one reviewer only, which could have resulted in some studies being missed. However, any doubts over inclusion/exclusion were discussed with a second reviewer before the final decision was made. In studies that included a diagnosis of dementia as a measure of cognitive impairment, this was based on data recorded in health insurance or patient registry databases, or patient, relative, or physician report. This may have led to an underestimation of the number of participants with dementia, an underestimation of those with at least some level of cognitive impairment, and subsequent underestimation of the association between dementia and (non)adherence. Further, there was substantial heterogeneity between studies, and only two of the included studies were considered as low risk of bias. Given the significant heterogeneity between studies in terms of assessments of medication adherence and cognitive impairment, and differential adjustment for confounders, the meta-analysis and sensitivity analyses should be interpreted with caution. Indeed, based on the GRADE assessment, the overall quality of the evidence was low, suggesting that the true association between cognitive impairment and non-adherence may be substantially different, with further research likely to have an impact on the estimates [31, 32]. In spite of these limitations however, the pooled estimates provide an important quantification of the substantial heterogeneity between the limited number of studies that have been published in this area, and highlight the need for further research, using clear, standardised and where possible objective assessments of both cognitive impairment and medication non-adherence.

### Conclusion

Few studies have explored associations between cognitive impairment and medication non-adherence in stroke patients. The substantial heterogeneity in study populations and definitions and assessments of adherence and cognitive impairment, coupled with the overall low quality of evidence, make it difficult to draw definitive conclusions. Given the importance of secondary prevention post-stroke and the association between medication adherence and outcomes, further research, with objective measures of adherence, is required to help identify those patients at greatest risk of non-adherence. Once suboptimal adherence has been recognised, care providers and patients can work together to address barriers to adherence and improve outcomes [11].

## Supporting information

S1 Table Database search terms. (DOCX)

S2 Table Studies excluded after full-text screening. (DOCX)

S1 Text PRISMA checklist. (DOC)

S2 Text PROSPERO protocol. (PDF)

S3 Text Cognitive impairment and adherence data. (XLS)
