# Supplementary material for: Does cognitive impairment impact adherence? A systematic review and meta-analysis of the association between cognitive impairment and medication non-adherence in stroke
Source: PLoS One. 2017 Dec 8;12(12):e0189339. doi: 10.1371/journal.pone.0189339 (PMC5722379; doi:10.1371/journal.pone.0189339)
Supplement: S1 Table — (DOCX) [file pone.0189339.s001.docx]

S1 Table. Database search terms

| **PubMed search terms** |
| --- |
| ((((((((stroke*[Title/Abstract]) OR ischem*[Title/Abstract]) OR ischaem*[Title/Abstract])) OR "Cerebrovascular Disorders"[Mesh])) |
| AND |
| ((((((dement*[Title/Abstract]) OR cogniti*[Title/Abstract]) OR memory[Title/Abstract]) OR alzheimer*[Title/Abstract])) OR (((("Dementia"[Mesh]) OR "Cognition Disorders"[Mesh:noexp]) OR "Mild Cognitive Impairment"[Mesh]) OR "Memory Disorders"[Mesh:noexp]))) |
| AND |
| ((((((((((((adhere*[Title/Abstract]) OR comply[Title/Abstract]) OR complies[Title/Abstract]) OR complian*[Title/Abstract]) OR non-complian*[Title/Abstract]) OR non-adhere*[Title/Abstract]) OR persist*[Title/Abstract]) OR non-persist*[Title/Abstract]) OR concordan*[Title/Abstract]) OR non-concordan*[Title/Abstract])) OR "Patient Compliance"[Mesh])) |
| AND |
| (((((((medication*[Title/Abstract]) OR drug*[Title/Abstract]) OR agent*[Title/Abstract]) OR treatment*[Title/Abstract]) OR therap*[Title/Abstract]) OR medicine*[Title/Abstract]) OR "Secondary Prevention"[Mesh]) |
| **Scopus search terms** |
| ( TITLE-ABS ( medication* OR drug* OR agent* OR treatment* OR therap* OR medicine* OR "secondary prevention" OR "secondary preventive" ) ) |
| AND |
| ( TITLE-ABS ( adhere* OR  comply OR complies OR complian* OR non-complian* OR non-adhere* OR persist* OR concordan* OR non-concordan* ) ) |
| AND |
| ( TITLE-ABS ( dement* OR cogniti* OR memory  OR alzheimer* ) ) |
| AND |
| ( TITLE-ABS ( stroke* OR ischem* OR ischaem* OR cerebrovascular ) ) |
| **PsycINFO search terms** |
| (medication* or drug* or agent* or treatment* or therap* or medicine* or "secondary prevention" or "secondary preventive").ab,ti. |
| AND |
| (treatment compliance.sh. OR compliance.sh.) OR ((adhere* or comply or complies or complian* or noncomplian* or non-adhere* or persist* or non-persist* or concordan* or non-concordan*).ab,ti.) |
| AND |
| (ischemia.sh. OR cerebral ischemia.sh. OR cerebrovascular disorders.sh. OR cerebrovascular accidents.sh.) OR ((stroke* or ischem* or ischaem*).ab,ti.) |
| AND |
| (dementia.sh. OR cognitive impairment.sh. OR memory disorders.sh. OR alzheimer's disease.sh. OR vascular dementia.sh.) OR ((dement* or cogniti* or memory or alzheimer*).ab,ti.) |
| *Final searches combined with AND .mp [mp=title, abstract, heading word, table of contents, key concepts, original title, tests & measures]* |
| **Embase search terms** |
| (‘secondary prevention’/exp AND [embase]/lim) OR (medication*:ab,ti OR drug*:ab,ti OR agent*:ab,ti OR treatment*:ab,ti OR therap*:ab,ti OR medicine*:ab,ti AND [embase]/lim) |
| AND |
| (‘patient compliance’/de AND [embase]/lim) OR (‘medication compliance’/exp AND [embase]/lim) OR (adhere*:ab,ti OR comply*:ab,ti OR complies*:ab,ti OR complian*:ab,ti OR ‘non compliant’*:ab,ti OR ‘non compliance’*:ab,ti) OR (non:ab,ti AND adherent:ab,ti) OR (non:ab,ti AND (adherence:ab,ti OR persist*:ab,ti)) OR (‘non persistence’:ab,ti OR ‘non persistent’:ab,ti OR concordan*:ab,ti OR ‘non concordance’:ab,ti OR ‘non concordant’:ab,ti) AND [embase]/lim |
| AND |
| (‘cognitive defect’/de AND [embase]/lim) OR (‘dementia’/exp AND [embase]/lim) OR (‘memory disorder’/de AND [embase]/lim) OR ‘mild cognitive impairment’/exp AND [embase]/lim) OR (dement*:ab,ti OR cogniti*:ab,ti OR memory:ab,ti OR Alzheimer:ab,ti AND [embase]/lim) |
| **Web of Science search terms** |
| (TI=(medication* OR drug* OR agent* OR treatment* OR therap* OR medicine* OR "secondary prevention" OR "secondary preventive")) OR (TS=(medication* OR drug* OR agent* OR treatment* OR therap* OR medicine* OR "secondary prevention" OR "secondary preventive")) |
| AND |
| (TI=(adhere* OR comply OR complies OR complian* OR non-complian* OR non-adhere* OR persist* OR non-persist* OR concordan* OR non-concordan*)) OR (TS=(adhere* OR comply OR complies OR complian* OR non-complian* OR non-adhere* OR persist* OR non-persist* OR concordan* OR non-concordan*)) |
| AND |
| (TI=(stroke* OR ischem* OR ischaem* OR cerebrovascular)) OR (TS=(stroke* OR ischem* OR ischaem* OR cerebrovascular)) |
| AND |
| (TI=(dement* OR cogniti* OR memory OR alzheimer*)) OR (TS=(dement* OR cogniti* OR memory OR alzheimer*)) |
| **Cochrane Library search terms** |
| ((medication* or drug* or agent* or treatment* or therap* or medicine*):ti,ab,kw (word variations have been search)) or (MeSH descriptor: [Secondary Prevention] explore all trees) |
| AND |
| ((adhere* or comply or complies or complian* or non-complian* or non-adhere* or persist* or non-persist* or concordan* or non-concordan*):ti,ab,kw (word variations have been searched)) or (MeSH descriptor: [Patient Compliance] explode all trees) |
| AND |
| ((dement* or cogniti* or memory or Alzheimer*):ti,ab,kw (Word variations have been searched)) or (MeSH descriptor: [Memory Disorders] this term only) or (MeSH descriptor: [Mild Cognitive Impairment] explode all trees) or (MeSH descriptor: [Dementia] explode all trees) or (MeSH descriptor: [Cognition Disorders] this term only) |
| AND |
| ((stroke* or ischem* or ischaem*):ti,ab,kw (word variations have been searched) or (MeSH descriptor: [Cerebrovascular Disorders] explode all trees) |
