# Supplementary material for: Does cognitive impairment impact adherence? A systematic review and meta-analysis of the association between cognitive impairment and medication non-adherence in stroke
Source: PLoS One. 2017 Dec 8;12(12):e0189339. doi: 10.1371/journal.pone.0189339 (PMC5722379; doi:10.1371/journal.pone.0189339)
Supplement: S2 Table — (DOCX) [file pone.0189339.s002.docx]

**S2 Table. Studies excluded after full-text screening**

| **No measure of cognition included or reported** |
| --- |
| Shaya FT, El Khoury AC, Mullins CD, Du D, Skolasky R, Fatodu H, et al. Drug therapy persistence and stroke recurrence. Am J Manag Care. 2006;12(6):313-9. |
| Polymeris AA, Traenka C, Hert L, Seiffge DJ, Peters N, De Marchis GM, et al. Frequency and Determinants of Adherence to Oral Anticoagulants in Stroke Patients with Atrial Fibrillation in Clinical Practice. Eur Neurol. 2016;76(3-4):187-93.10.1159/000450750. |
| Bushnell CD, Olson DM, Zhao X, Pan W, Zimmer LO, Goldstein LB, et al. Secondary preventive medication persistence and adherence 1 year after stroke. Neurol. 2011;77(12):1182-90.10.1212/WNL.0b013e31822f0423. |
| Burke JP, Sander S, Shah H, Zarotsky V, Henk H. Impact of persistence with antiplatelet therapy on recurrent ischemic stroke and predictors of nonpersistence among ischemic stroke survivors. Curr Med Res Opin. 2010;26(5):1023-30.10.1185/03007991003670563. |
| Hamann GF, Weimar C, Glahn J, Busse O, Diener HC. Adherence to secondary stroke prevention strategies--results from the German Stroke Data Bank. Cerebrovasc Dis. 2003;15(4):282-8.69490. |
| Hillen T, Dundas R, Lawrence E, Stewart JA, Rudd AG, Wolfe CD. Antithrombotic and antihypertensive management 3 months after ischemic stroke : a prospective study in an inner city population. Stroke. 2000;31(2):469-75 |
| Hohmann C, Neumann-Haefelin T, Klotz JM, Freidank A, Radziwill R. Adherence to hospital discharge medication in patients with ischemic stroke: a prospective, interventional 2-phase study. Stroke. 2013;44(2):522-4.10.1161/strokeaha.112.678847. |
| Ji R, Liu G, Shen H, Wang Y, Li H, Peterson E, et al. Persistence of secondary prevention medications after acute ischemic stroke or transient ischemic attack in Chinese population: data from China National Stroke Registry. Neurol Res. 2013;35(1):29-36.10.1179/1743132812y.0000000107. |
| Murphy SJ, Coughlan CA, Tobin O, Kinsella J, Lonergan R, Gutkin M, et al. Continuation and adherence rates on initially-prescribed intensive secondary prevention therapy after Rapid Access Stroke Prevention (RASP) service assessment. J Neurol Sci. 2016;361:13-8.10.1016/j.jns.2015.12.009. |
| Ovbiagele B, Saver JL, Fredieu A, Suzuki S, Selco S, Rajajee V, et al. In-hospital initiation of secondary stroke prevention therapies yields high rates of adherence at follow-up. Stroke. 2004;35(12):2879-83.10.1161/01.STR.0000147967.49567.d6. |
| Phillips LA, Diefenbach MA, Abrams J, Horowitz CR. Stroke and TIA survivors' cognitive beliefs and affective responses regarding treatment and future stroke risk differentially predict medication adherence and categorised stroke risk. Psychol Health. 2015;30(2):218-32.10.1080/08870446.2014.964237. |
| Sappok T, Faulstich A, Stuckert E, Kruck H, Marx P, Koennecke HC. Compliance with secondary prevention of ischemic stroke: a prospective evaluation. Stroke. 2001;32(8):1884-9. |
| Sauer R, Sauer EM, Bobinger T, Blinzler C, Huttner HB, Schwab S, et al. Adherence to oral anticoagulation in secondary stroke prevention--the first year of direct oral anticoagulants. J Stroke Cerebrovasc Dis. 2015;24(1):78-82.10.1016/j.jstrokecerebrovasdis.2014.07.032. |
| Colivicchi F, Bassi A, Santini M, Caltagirone C. Discontinuation of statin therapy and clinical outcome after ischemic stroke. Stroke. 2007;38(10):2652-7.10.1161/strokeaha.107.487017. |
| Kamal AK, Shaikh Q, Pasha O, Azam I, Islam M, Memon AA, et al. A randomized controlled behavioral intervention trial to improve medication adherence in adult stroke patients with prescription tailored Short Messaging Service (SMS)-SMS4Stroke study. BMC Neurol. 2015;15:212.10.1186/s12883-015-0471-5. |
| Weimar C, Benemann J, Katsarava Z, Weber R, Diener HC. Adherence and quality of oral anticoagulation in cerebrovascular disease patients with atrial fibrillation. Eur Neurol.  2008;60(3):142-8.10.1159/000144085. |
| **Association between cognitive function and medication adherence not available** |
| Lummis HL, Sketris IS, Gubitz GJ, Joffres MR, Flowerdew GJ. Medication persistence rates and factors associated with persistence in patients following stroke: a cohort study. BMC Neurol. 2008;8:25.10.1186/1471-2377-8-25. |
| Chausson N, Olindo S, Cabre P, Saint-Vil M, Smadja D. Five-year outcome of a stroke cohort in Martinique, French West Indies: Etude Realisee en Martinique et Centree sur l'Incidence des Accidents vasculaires cerebraux, Part 2. Stroke. 2010;41(4):594-9.10.1161/strokeaha.109.573402. |
| Ireland SE, Arthur HM, Gunn EA, Oczkowski W. Stroke prevention care delivery: predictors of risk factor management outcomes. Int J Nurs Stud. 2011;48(2):156-64.10.1016/j.ijnurstu.2010.07.003. |
| Luzzi A, Alonzo C, Brescacin L, Zurru M, Pigretti S, Machado PC, et al. Two-year adherence to a stroke prevention program in a Latin American cohort. Neurol. 2015;84 |
| Mackenzie G, Ireland S, Moore S, Heinz I, Johnson R, Oczkowski W, et al. Tailored interventions to improve hypertension management after stroke or TIA--phase II (TIMS II). Can J Neurosci Nurs. 2013;35(1):27-34 |
| **General adult populations with sub-group analyses for patients with stroke not available** |
| Wilke T, Groth A, Pfannkuche M, Harks O, Fuchs A, Maywald U, et al. Real life anticoagulation treatment of patients with atrial fibrillation in Germany: extent and causes of anticoagulant under-use. J Thromb Thrombolysis. 2015;40(1):97-107.10.1007/s11239-014-1136-8. |
| Vinyoles E, De la Figuera M, Gonzalez-Segura D. Cognitive function and blood pressure control in hypertensive patients over 60 years of age: COGNIPRES study. Curr Med Res Opin. 2008;24(12):3331-9.10.1185/03007990802538724. |
| Lapi F, Lucenteforte E, Bonaiuti R, Moschini M, Pugi A, Di Pirro M, et al. Validation of 'fiesole misurata' database: An ongoing project to investigate how to enhance adherence to antihypertensive medications (AM). Basic and Clinical Pharmacology and Toxicology. 2011;109:107. |
| Tajeu GS, Kent ST, Kronish IM, Huang L, Krousel-Wood M, Bress AP, et al. Trends in Antihypertensive Medication Discontinuation and Low Adherence Among Medicare Beneficiaries Initiating Treatment From 2007 to 2012. Hypertension. 2016;68(3):565-75.10.1161/hypertensionaha.116.07720. |
| Leung DYP, Bai X, Leung AY, Liu BC, Chi I. Prevalence of medication adherence and its associated factors among community-dwelling Chinese older adults in Hong Kong. Geriatrics and Gerontology International. 2015;15(6):789-96 |
