# Supplementary material for: Does cognitive impairment impact adherence? A systematic review and meta-analysis of the association between cognitive impairment and medication non-adherence in stroke
Source: PLoS One. 2017 Dec 8;12(12):e0189339. doi: 10.1371/journal.pone.0189339 (PMC5722379; doi:10.1371/journal.pone.0189339)
Supplement: S2 Text — (PDF) [file pone.0189339.s004.pdf]

PROSPERO International prospective register of systematic reviews

Review title and timescale

1 Review title

Give the working title of the review. This must be in English. Ideally it should state succinctly the interventions or exposures being reviewed and the associated health or social problem being addressed in the review.

Systematic review of the association between adherence to secondary preventive medications and cognitive impairment in stroke patients

2 Original language title

For reviews in languages other than English, this field should be used to enter the title in the language of the review. This will be displayed together with the English language title.

3 Anticipated or actual start date

Give the date when the systematic review commenced, or is expected to commence.

02/11/2015

4 Anticipated completion date

Give the date by which the review is expected to be completed.

31/07/2017

5 Stage of review at time of this submission

Indicate the stage of progress of the review by ticking the relevant boxes. Reviews that have progressed beyond the point of completing data extraction at the time of initial registration are not eligible for inclusion in PROSPERO. This field should be updated when any amendments are made to a published record.

The review has not yet started ×

Review stage	Started	Completed
Preliminary searches	No	Yes
Piloting of the study selection process	No	Yes
Formal screening of search results against eligibility criteria	Yes	Yes
Data extraction	Yes	Yes
Risk of bias (quality) assessment	No	Yes
Data analysis	No	Yes

Provide any other relevant information about the stage of the review here.

Review team details

6 Named contact

The named contact acts as the guarantor for the accuracy of the information presented in the register record.

Ms Rohde

7 Named contact email

Enter the electronic mail address of the named contact.

danielamrohde@rcsi.ie

8 Named contact address

Enter the full postal address for the named contact.

Department of Psychology, Division of Population Health Sciences, Royal College of Surgeons in Ireland, Dublin 2, Ireland

9 Named contact phone number

Enter the telephone number for the named contact, including international dialing code.

+353 1 402 8622

10 Organisational affiliation of the review

Full title of the organisational affiliations for this review, and website address if available. This field may be completed

as 'None' if the review is not affiliated to any organisation.
Royal College of Surgeons in Ireland (RCSI)

Website address:
www.rcsi.ie

11 Review team members and their organisational affiliations
Give the title, first name and last name of all members of the team working directly on the review. Give the organisational affiliations of each member of the review team.

Title	First name	Last name	Affiliation
Ms	Daniela	Rohde	Royal College of Surgeons in Ireland
Professor	Anne	Hickey	Royal College of Surgeons in Ireland
Professor	David	Williams	Royal College of Surgeons in Ireland & Beaumont Hospital
Dr	Kathleen	Bennett	Royal College of Surgeons in Ireland
Dr	NIamh A.	Merriman	Royal College of Surgeons in Ireland
Dr	Frank	Doyle	Royal College of Surgeons in Ireland

12 Funding sources/sponsors
Give details of the individuals, organizations, groups or other legal entities who take responsibility for initiating, managing, sponsoring and/or financing the review. Any unique identification numbers assigned to the review by the individuals or bodies listed should be included.
This work was supported by the Irish Health Research Board (grant nos. SPHeRE 2013/1 and RL-15-1579).

13 Conflicts of interest
List any conditions that could lead to actual or perceived undue influence on judgements concerning the main topic investigated in the review.
Are there any actual or potential conflicts of interest?
Yes

FD has accepted an honorarium from Abbvie for speaking on the topic of medication adherence. DW reports personal fees from Bayer, personal fees from Boehringer Ingelheim, personal fees from Bristol Myers Squibb, and personal fees from Daiichi Sankyo, outside the submitted work. All other authors declare that they have no conflicts of interest.

14 Collaborators
Give the name, affiliation and role of any individuals or organisations who are working on the review but who are not listed as review team members.

Title	First name	Last name	Organisation details
Ms	Grainne	McCabe	RCSI

Review methods

15 Review question(s)
State the question(s) to be addressed / review objectives. Please complete a separate box for each question.
1. What is the impact of adherence to secondary preventive medications on subsequent cognitive impairment in stroke?

2. What is the impact of cognitive impairment on subsequent medication (non)adherence in stroke?

16 Searches
Give details of the sources to be searched, and any restrictions (e.g. language or publication period). The full search strategy is not required, but may be supplied as a link or attachment.
The following electronic bibliographic databases were searched without restrictions: PubMed, EMBASE, PsycINFO, Web of Science, Scopus and the Cochrane Library. Search strategies were developed in consultation with a librarian. Search strategies for PubMed and Scopus are listed below. PubMed: ((((((((stroke*[Title/Abstract]) OR ischem*[Title/Abstract]) OR ischaem*[Title/Abstract])) OR "Cerebrovascular Disorders"[Mesh])) AND

((((((dement*[Title/Abstract]) OR cogniti*[Title/Abstract]) OR memory[Title/Abstract]) OR alzheimer*[Title/Abstract])) OR (((("Dementia"[Mesh]) OR "Cognition Disorders"[Mesh:noexp]) OR "Mild Cognitive Impairment"[Mesh]) OR "Memory Disorders"[Mesh:noexp]))) AND ((((((((((((adhere*[Title/Abstract]) OR comply[Title/Abstract]) OR complies[Title/Abstract]) OR complian*[Title/Abstract]) OR non-complian*[Title/Abstract]) OR non-adhere*[Title/Abstract]) OR persist*[Title/Abstract]) OR non-persist*[Title/Abstract]) OR concordan*[Title/Abstract]) OR non-concordan*[Title/Abstract])) OR "Patient Compliance"[Mesh])) AND (((((((medication*[Title/Abstract]) OR drug*[Title/Abstract]) OR agent*[Title/Abstract]) OR treatment*[Title/Abstract]) OR therap*[Title/Abstract]) OR medicine*[Title/Abstract]) OR "Secondary Prevention"[Mesh]) Scopus: (TITLE-ABS(medication* OR drug* OR agent* OR treatment* OR therap* OR medicine* OR "secondary prevention" OR "secondary preventive")) AND (TITLE-ABS(adhere* OR comply OR complies OR complian* OR non-complian* OR non-adhere* OR persist* OR concordan* OR non-concordan*)) AND (TITLE-ABS(dement* OR cogniti* OR memory OR alzheimer*)) AND (TITLE-ABS(stroke* OR ischem* OR ischaem* OR cerebrovascular)) Reference lists of eligible studies will also be searched.

17 URL to search strategy

If you have one, give the link to your search strategy here. Alternatively you can e-mail this to PROSPERO and we will store and link to it.

I give permission for this file to be made publicly available
Yes

18 Condition or domain being studied

Give a short description of the disease, condition or healthcare domain being studied. This could include health and wellbeing outcomes.
Stroke. Cognitive impairment. Medication adherence.

19 Participants/population

Give summary criteria for the participants or populations being studied by the review. The preferred format includes details of both inclusion and exclusion criteria.
Inclusion criteria: Adults with stroke, as diagnosed using any recognised diagnostic criteria. Exclusion criteria: Children and adolescents (under 18 years of age); adults without stroke

20 Intervention(s), exposure(s)

Give full and clear descriptions of the nature of the interventions or the exposures to be reviewed
1. A variety of methods have been used to assess medication (non)adherence, including self-report, pill counts, electronic pill bottles, and pharmacy prescription refills. Studies reporting any measure of medication (non)adherence will be included. 2. Cognitive impairment in stroke patients may be assessed using a variety of measures, including assessments such as the Montreal Cognitive Assessment (MoCA), Mini Mental State Exam (MMSE), or comprehensive neuropsychological test batteries. Studies reporting any measure of cognitive function will be included.

21 Comparator(s)/control

Where relevant, give details of the alternatives against which the main subject/topic of the review will be compared (e.g. another intervention or a non-exposed control group).
1. In determining the impact of medication adherence on cognitive function, stroke patients with good adherence (usually defined as >=80%) will be compared to patients with poor adherence on measures of cognitive impairment. 2. In order to assess the impact of cognitive impairment on medication adherence, stroke patients without cognitive impairment will be compared to stroke patients with cognitive impairment on adherence to medications.

22 Types of study to be included

Give details of the study designs to be included in the review. If there are no restrictions on the types of study design eligible for inclusion, this should be stated.
Inclusion: Longitudinal (cohort, (non)randomised controlled trials, case-control) or cross-sectional studies. Exclusion: Reviews, letters, editorials, qualitative studies.

23 Context

Give summary details of the setting and other relevant characteristics which help define the inclusion or exclusion criteria.
1. In order to assess the impact of adherence to secondary preventive medications on subsequent cognitive impairment in stroke patients, longitudinal studies reporting a measure of medication adherence at baseline and cognitive impairment at follow-up will be included. 2. In order to assess the impact of cognitive impairment on

subsequent medication (non)adherence, longitudinal studies reporting a measure of cognitive impairment at baseline and medication adherence at follow-up will be included. Cross-sectional studies reporting the association between cognitive impairment and medication adherence at one time will also be included, with results synthesised separately.

24 Primary outcome(s)

Give the most important outcomes.

1. Cognitive impairment in stroke patients may be assessed using a variety of measures, including the Montreal Cognitive Assessment (MoCA), Mini Mental State Exam (MMSE), or comprehensive neuropsychological test batteries. Studies reporting any measure of cognitive impairment at follow-up will be included. For studies reporting measures of cognitive impairment at both baseline and follow-up, decline in cognitive function will be considered as a decrease in score from baseline to follow-up. 2. Medication (non)adherence may be assessed using a variety of methods, including self-report, pill counts, electronic pill bottles, and pharmacy prescription refills. Studies reporting any measure of medication (non)adherence at follow-up will be included.

Give information on timing and effect measures, as appropriate.

25 Secondary outcomes

List any additional outcomes that will be addressed. If there are no secondary outcomes enter None.

None

Give information on timing and effect measures, as appropriate.

26 Data extraction (selection and coding)

Give the procedure for selecting studies for the review and extracting data, including the number of researchers involved and how discrepancies will be resolved. List the data to be extracted.

All records retrieved using search strategies and those identified from additional sources were initially imported into EndNote to identify duplicate records. The remaining records were then exported to Covidence. These records will be screened independently by two reviewers to identify studies that potentially meet the inclusion criteria. Full texts of potentially eligible studies will be retrieved and independently assessed for eligibility by two reviewers. Any disagreements will be resolved through discussion with a third member of the review team. Data from the included studies will be extracted using a standardised, pre-piloted data extraction form. Two reviewers will extract data independently, with discrepancies identified and resolved through discussion, with a third author if necessary. Extracted information will include: authors, study design, sample size (baseline and follow-up), sample description, length of follow-up, measure of medication adherence, measure of cognitive impairment, statistical results (adjusted and unadjusted), comments, and study conclusions. Study authors will be contacted for missing data or further information if necessary.

27 Risk of bias (quality) assessment

State whether and how risk of bias will be assessed, how the quality of individual studies will be assessed, and whether and how this will influence the planned synthesis.

Risk of bias will be assessed independently by two reviewers using the Cochrane Bias Methods Group's Tool to Assess Risk of Bias in Cohort Studies. This checklist assesses the risk of bias, from low to high, for key aspects of each study, including sample selection, assessment of exposure and outcome, presence/absence of outcome at the beginning of the study, assessment of and adjustment for prognostic variables, and follow-up. Disagreements between review authors over the risk of bias in particular studies will be resolved through discussion, with a third author if necessary. No study will be excluded as a result of findings from the risk of bias assessments. However, if substantial variation in risk of bias of included studies is found, results will be synthesised separately for studies at high risk and low risk of bias.

28 Strategy for data synthesis

Give the planned general approach to be used, for example whether the data to be used will be aggregate or at the level of individual participants, and whether a quantitative or narrative (descriptive) synthesis is planned. Where appropriate a brief outline of analytic approach should be given.

Due to substantial heterogeneity in assessments and classification of cognitive impairment and medication adherence, a qualitative narrative synthesis will be conducted, structured around the two outcomes of interest (cognitive impairment and medication adherence), and study characteristics (study design (cross-sectional or longitudinal), length of follow-up, risk of bias etc.). However, it may also be possible to standardise outcome scores; If the standardised outcomes demonstrate homogeneity, results for individual outcomes will be pooled quantitatively using meta-analysis. Studies reporting the cross-sectional associations between cognitive impairment and medication

adherence may also be pooled quantitatively using meta-analysis.

29 Analysis of subgroups or subsets
Give any planned exploration of subgroups or subsets within the review. 'None planned' is a valid response if no subgroup analyses are planned.
A planned subgroup analysis for the qualitative synthesis will be undertaken for cross-sectional and longitudinal studies. While other subgroup analyses may also be undertaken (e.g. first-ever vs. recurrent stroke), it is not possible to specify these groups in advance. Depending on the available data, quantitative sensitivity analyses may be possible. These could explore different lengths of follow-up, first-ever vs. recurrent stroke, different types of assessments used, hospital vs. population-based samples, different study designs, etc.

Review general information

30 Type and method of review
Select the type of review and the review method from the drop down list.
Meta-analysis, Systematic review

Neurological

31 Language
Select the language(s) in which the review is being written and will be made available, from the drop down list. Use the control key to select more than one language.
English

Will a summary/abstract be made available in English?
Yes

32 Country
Select the country in which the review is being carried out from the drop down list. For multi-national collaborations select all the countries involved. Use the control key to select more than one country.
Ireland

33 Other registration details
Give the name of any organisation where the systematic review title or protocol is registered together with any unique identification number assigned. If extracted data will be stored and made available through a repository such as the Systematic Review Data Repository (SRDR), details and a link should be included here.

34 Reference and/or URL for published protocol
Give the citation for the published protocol, if there is one.
Give the link to the published protocol, if there is one. This may be to an external site or to a protocol deposited with CRD in pdf format.

I give permission for this file to be made publicly available
Yes

35 Dissemination plans
Give brief details of plans for communicating essential messages from the review to the appropriate audiences.
A paper based on this review will be submitted to a journal in this field.

Do you intend to publish the review on completion?
Yes

36 Keywords
Give words or phrases that best describe the review. (One word per box, create a new box for each term)
stroke

medication adherence

cognitive impairment

37 Details of any existing review of the same topic by the same authors
Give details of earlier versions of the systematic review if an update of an existing review is being registered, including full bibliographic reference if possible.

38 Current review status
Review status should be updated when the review is completed and when it is published.
Completed but not published

39 Any additional information
Provide any further information the review team consider relevant to the registration of the review.

40 Details of final report/publication(s)
This field should be left empty until details of the completed review are available.
Give the full citation for the final report or publication of the systematic review.
Give the URL where available.
